# Comprehensive Analysis of Respiratory Burst Oxidase Homologs (Rboh) Gene Family and Function of *GbRboh5/18* on Verticillium Wilt Resistance in *Gossypium barbadense*

**DOI:** 10.3389/fgene.2020.00788

**Published:** 2020-09-11

**Authors:** Ying Chang, Bo Li, Qian Shi, Rui Geng, Shuaipeng Geng, Jinlei Liu, Yuanyuan Zhang, Yingfan Cai

**Affiliations:** State Key Laboratory of Cotton Biology, Key Laboratory of Plant Stress Biology, School of Life Sciences, Henan University, Kaifeng, China

**Keywords:** respiratory burst oxidase homologs (Rbohs), reactive oxygen species (ROS), *Gossypium*, *V. dahliae*, *GbRboh5/18*

## Abstract

Respiratory burst oxidase homologs (Rbohs) play a predominant role in reactive oxygen species (ROS) production, which is crucial in plant growth, differentiation, as well as their responses to biotic and abiotic stresses. To date, however, there is little knowledge about the function of cotton *Rboh* genes. Here, we identified a total of 87 *Rbohs* from five sequenced *Gossypium* species (the diploids *Gossypium arboreum*, *Gossypium raimondii*, and *Gossypium australe*, and the allotetraploids *Gossypium hirsutum* and *Gossypium barbadense*) via BLAST searching their genomes. Phylogenetic analysis of the putative 87 cotton *Rbohs* revealed that they were divided into seven clades. All members within the same clade are generally similar to each other in terms of gene structure and conserved domain arrangement. In *G. barbadense*, the expression levels of *GbRbohs* in the CladeD were induced in response to a fungal pathogen and to hormones (i.e., jasmonic acid and abscisic acid), based upon which the main functional member in CladeD was discerned to be *GbRboh5/18.* Further functional and physiological analyses showed that the knock-down of *GbRboh5/18* expression attenuates plant resistance to *Verticillium dahliae* infection. Combined with the molecular and biochemical analyses, we found less ROS accumulation in *GbRboh5/18-VIGS* plants than in control plants after *V. dahliae* infection. Overexpression of *GbRboh5/18* in *G. barbadense* resulted in more ROS accumulation than in control plants. These results suggest that *GbRboh5/18* enhances the cotton plants’ resistance against *V. dahliae* by elevating the levels of ROS accumulation. By integrating phylogenetic, molecular, and biochemical approaches, this comprehensive study provides a detailed overview of the number, phylogeny, and evolution of the *Rboh* gene family from five sequenced *Gossypium* species, as well as elucidating the function of *GbRboh5/18* for plant resistance against *V. dahliae*. This study sheds fresh light on the molecular evolutionary properties and function of *Rboh* genes in cotton, and provides a reference for improving cotton’s responses to the pathogen *V. dahliae*.

## Introduction

Reactive oxygen species (ROS) play dual roles in plant growth, and development, and in their responses to biotic and abiotic stresses (Torres and Dangl, 2005; Suzuki et al., 2011). Hydrogen peroxide (H_2_O_2_), the most stable species of ROS, can serve as a secondary signaling molecule for transduction in the cytoplasmic at a low basal level. However, because of the damage they cause to plant cell’s DNA, proteins, and lipids, ROS species are toxic when the accumulation exceeds a certain threshold, which can occur for the superoxide anion radical (O_2_^–^), hydroxyl radical (⋅OH), and H_2_O_2_ (Baxter et al., 2014).

Respiratory burst oxidase homologs (Rbohs), which are plant-specific NADPH oxidases, are key enzymes that move electrons from cytoplasmic NADPH to O_2_ and thus foster the generation of O_2_^–^, followed by the dismutation of O_2_^–^ into H_2_O_2_ (Torres et al., 1998; Sagi and Fluhr, 2006). The first plant *Rboh* gene was found in rice, named *OsRbohA*, for which the mammalian homolog is *gp91*^phox^/*Nox2* (Keller et al., 1998). Then more Rbohs from plants were successively identified from model plants (including Arabidopsis and tobacco) and crops, such as tomato, potato, and cucumber (Torres et al., 1998; Yoshioka et al., 2003; Sagi et al., 2004; Kobayashi et al., 2006; Xia et al., 2009; Yu et al., 2019). Generally, plant Rboh proteins are best described as integral plasma membrane proteins containing six transmembrane (TM) domains. The heme groups usually bind two pairs of histidine residues, respectively located in third and fifth TMs, to facilitate electron transport. Two Ca^2+^-binding EF-hand motifs and phosphorylation sites in the cytological N-terminal region participate in regulating enzymatic activity. Similar to the mammalian *gp91*^phox^, there are flavin adenine dinucleotide (FAD) and NADPH-binding domains on the C-terminus of Rboh proteins in plants (Wong et al., 2007).

Ten and nine Rbohs have been identified from Arabidopsis and rice, referred to as AtRbohA–AtRbohJ and OsRbohA–OsRbohI, respectively (Torres et al., 1998). Previous studies suggest they have distinct spatio-temporal expression, enzyme activity, and functional diversification (Lin et al., 2009; Kaur and Pati, 2018; Kaur et al., 2018; Kaya et al., 2019). *AtrbohB* fulfills important roles in the germination of Arabidopsis seeds through variant splicing transcripts (Muller et al., 2009). *AtRbohD* and *AtRbohF* present a different spatial expression pattern either in shoot or in root during Arabidopsis development (Jiao et al., 2013; Morales et al., 2016). Finally, AtRbohC, -D, -F, -H, and -J are synergistically activated by Ca^2+^-binding and then phosphorylated to produce ROS (Ogasawara et al., 2008; Han et al., 2019; Kaya et al., 2019).

Previous studies have proven that Rbohs figure predominantly in ROS production during pathogen-associated molecular pattern-triggered immunity (PTI) and effector-triggered immunity (ETI) responses in Arabidopsis (Torres and Dangl, 2005; Nuhse et al., 2007; Kadota et al., 2014; Li et al., 2014b). Perhaps the best example is RbohD (and RbohF in some cases), a key player not only in intracellular oxidative stresses but also in plant responses and resistance to pathogens (Yun et al., 2011; Chaouch et al., 2012; Ma et al., 2016; Angelos and Brandizzi, 2018). AtRbohD showed the highest expression of the 10 *AtRbohs*: it functions via ROS production in abscisic acid-induced stomatal closure, flagellin-induced immune responses (Pogany et al., 2009), resistance to *Botrytis cinereal* (Galletti et al., 2008), as well as phosphorylation mediated by CRK2 in plant defense against the bacterial pathogen *Pseudomonas syringae* pv. tomato DC3000 (Kimura et al., 2020). AtRbohD-dependent H_2_O_2_ production was positively regulated by H2Bub1 in the responses against Vd-toxins in Arabidopsis (Zhao et al., 2020). DORN-mediated phosphorylation on the N-terminus of RbohD led to ROS accumulation and stomatal closure of Arabidopsis in biotic stresses (Chen et al., 2017). NbrbohB participates in H_2_O_2_ accumulation, resulting in elicitor-induced stomatal closure, but it does not function in elicitor-induced HR in *Nicotiana benthamiana* (Yoshioka et al., 2003; Kobayashi et al., 2007; Zhang et al., 2009). Ethylene signaling can regulate ROS production and enhance disease resistance, in which both are mediated by OsEIL2 binding to the promoter of *OsrbohA* and *OsrbohB* in rice (Yang et al., 2017).

Cotton (*Gossypium* spp.) is a globally important fiber crop that is the largest source of natural renewable textile fibers. The allotetraploids *Gossypium hirsutum* and *Gossypium barbadense* are cultivated worldwide because of their high yield and superior fiber quality, respectively (Wendel, 1989; Paterson et al., 2012; Wang et al., 2019). Both *G. hirsutum* and *G. barbadense* originated from two diploid progenitor species: *Gossypium arboreum* (A-genome donor) and *Gossypium raimondii* (D-genome donor) ca. 1–2 million years ago (Wendel, 1989). Because of their tetraploidy and repeat sequences, *G. hirsutum* and *G. barbadense* have large genomes; hence, their study from a genetics and genomics perspective is quite difficult (Paterson et al., 2012; Li et al., 2014a, 2015; Zhang et al., 2015). Moreover, the wild diploid cotton species *Gossypium australe* possesses excellent traits for plant resistance to disease, and its genome was fully sequenced and assembled last year (Cai et al., 2019). Nevertheless, with recent advances in sequencing technology and high-quality genome assemblies and annotations, it is much easier now to pursue detailed analyses of plant genomics (Wang et al., 2019).

Verticillium wilt, caused by the soil-born fungus *Verticillium dahliae* Kleb., is a serious widespread disease that can severely affect plant growth and fiber quality in cotton production fields (Shaban et al., 2018; Wang et al., 2018). The disease symptoms of *V. dahliae* infection consists of necrotic areas on leaves, yellowing of leaves, wilting and defoliation, and discoloration of vascular tissues (Li et al., 2017). Currently, verticillium wilt has yet to be controlled effectively by either genetic means or fungicide applications, so analyzing the resistance mechanisms of cotton against verticillium wilt should help to resolve it. Although some studies have investigated the function of Rbohs in biotic stress defense responses of plants, detailed genome-wide phylogenetic and functional analyses of the *Rboh* genes family in *G. barbadense* are still lacking.

To better understand the dynamics of *Rboh* genes in *Gossypium* plants and to foster future research on this important enzyme family, here we aimed to provide a detailed overview of their number, phylogeny, and function in defense against *V. dahliae*, by investigating five sequenced *Gossypium* species. From these, we identified 87 *Rbohs*. We determined that not only did *GbRboh5/18* in the CladeD respond to *V. dahliae* infection and hormones, but it was also able to enhance plant resistance against verticillium wilt through elevated ROS accumulation in *G*. *barbadense*.

## Materials and Methods

### Sequence Retrieval and Identification of Cotton *Rboh* Genes

The motif FAD_binding_8 (PF08022) and EF-hand 2 (PF09068) were each downloaded from the Pfam database^1^. The whole genome sequence from the ancestral diploids *G. arboreum* (A_2_) (Li et al., 2014a; Du et al., 2018) and *G. raimondii* (D_5_) (Paterson et al., 2012), as well as those of their descendant tetraploids, *G. barbadense* (AD_2_) (Wang et al., 2019), and *G. hirsutum* (AD_1_) (Li et al., 2015; Zhang et al., 2015; Wang et al., 2019) were downloaded from the CottonGen database^2^, while that of *G. australe* (G) (Cai et al., 2019) was downloaded from NCBI^3^ (accession number: SMMG00000000), to carry out a genome-wide search for Rboh genes in the *Gossypium* genus. Splice variants were excluded and only the first variant was retained for further analysis. The molecular weights (kDa) and isoelectric points (*p*I) of Rboh proteins were determined using ProtParam^4^, and the subcellular localization of each protein was predicted by SherLoc2^5^.

### Phylogenetic, Gene Structure and Conserved Domain Analysis

A total of 10 Arabidopsis *Rboh* gene sequences were downloaded from TAIR 10^6^. Multiple sequence alignments of all identified Rbohs from cotton and Arabidopsis were performed in ClustalX 2.0. The phylogenetic tree of deduced amino acid sequences was constructed by applying the neighbor-joining (NJ) method, with default parameters and 1000 bootstrap replicates, in MEGA7.0^7^. To analyze the exon-intron distributions of the *Rboh* gene, the gene structure display server (GSDS)^8^ was utilized. Conserved motifs were then predicted using the MEME^9^ tool, under these parameters: number of repetitions, “any”; maximum number of motifs, “20”; and 60≥ “widths” ≥5, and between 6 and 300 residues. Only those motifs associated with an *E*-value < e^–5^ were retained.

### Chromosomal Localization

The positional information of a given cotton *Rboh* gene was obtained from parsed general feature format (GFF) files, downloaded from the CottonGen website. The *Rbohs* in the five *Gossypium* species genomes were all mapped onto the chromosome. A shared identity of genes between different *Gossypium* spp. above 91%, or an identity of genes between the A and D subgenomes on tetraploid *Gossypium* spp. higher than 98% were connected by different-colored lines and depicted with the Circos genome visualization tool^10^.

### Plant Materials, Growth Condition, and Treatments

Unless otherwise indicated, seeds of *G. barbadense* cultivar (“Xinhai15”) were inoculated in ddH_2_O for 24 h and then germinated for another 24 h under 25°C. After they had absorbed water, two seeds were sown per pot containing vermiculite. The pots were kept on a tray covered with a plastic dome, in a growth chamber at 25°C, with 120 μE m^–2^ s^–1^ light illumination under a 12-h-light/12-h-dark photoperiod. The domes were removed when two cotyledons emerged on all seedlings. When the first two true leaves fully expanded and the third true leaf emerged, the cotton plants could be treated with *V. dahliae* or hormones. For the former, the *V. dahliae* suspension was injected into the vermiculite nearby root; for the latter, plants were sprayed with jasmonic acid (JA; 100 μM concentration) or with abscisic acid (ABA; 50 μM concentration).

### RNA Extraction, Reverse Transcription and Quantitative Real-Time PCR Analyses

Total RNA was extracted from cotton leaves using the RNAprep Pure Plant Plus Kit (DP441, Tiangen). Reverse transcription was performed with the HiScript II Q Select RT SuperMix (R233, Vazyme Biotech, Nanjing, China) following the manufacturer’s instructions. The quantitative PCR reactions employed the ChamQ SYBR Color qPCR Master Mix (Q411-02, Vazyme Biotech) and were run on the LightCycler480II Real-time PCR system (Roche) (every Q-PCR reaction was carried in triplicate for each sample). The cotton *UBQ7* gene served as an internal reference gene for the normalization of the data. The sequences of all primer used can be found in Supplementary Table S2. For a given sample, the gene’s Ct values were standardized and its relative changes analyzed using the 2^–ΔΔCT^ method.

### Vector Construction

The *TRV-VIGS* vectors were constructed as described previously (Liu et al., 2002). The CDS fragment of *GbRboh5/18* (Gbar_A05G025370/Gbar_D05G026620) and the fragment of 297 bp for virus-induced gene silencing (VIGS) with *Bam*HI/*Kpn*I at the 5′ and 3′ ends, were amplified from *G. barbadense* L. “Xinhai15” cDNA by PCR with gene-specific primers (listed in Supplementary Table S2) and cloned into the *pMD18*-T vector. The *GbRboh5/18-VIGS* fragment was inserted into the *pTRV2* vector by *Bam*HI/*Kpn*I. All plasmids (including empty vector *pTRV1*, *pTRV2*, and recombinant vectors *pTRV2*- *GbRboh5/18*, *pTRV2-CLA* as the positive control) were respectively electroporated into the *Agrobacterium tumefaciens* strain GV3101, which was used to inject cotton cotyledons for VIGS.

The full length CDS fragment of *GbRboh5/18* was amplified from *G. barbadense* L. “Xinhai15” cDNA by PCR with specific primers (listed in Supplementary Table S2). The overexpression vector was constructed using the Gateway Technology (Invitrogen). The PCR product was first conducted in BP reaction with *pDONOR221* vector. Then, the positive plasmid *pDONOR221-GbRboh5/18* underwent an LR reaction with the *pK7WG2.0* vector to generate the *p35S::GbRboh5/18* construct. The positive plasmid *pK7WG2.0-GbRboh5/18* was electroporated into *A. tumefaciens* strain LBA4404, which was used to transform cotton plants for transient overexpression.

### Virus-Induced Gene Silencing of *GbRboh5/18* in Cotton

Virus-induced gene silencing assays were performed as described previously (Li et al., 2016) with minor modifications. The cotton plants with two fully expanded cotyledons were selected for infiltration for the VIGS assay. The *A. tumefaciens* strain GV3101 cells carrying the corresponding plasmid were inoculated into an LB liquid medium (containing 50 μg mL^–1^ kanamycin, 50 μg mL^–1^ rifampicin, and 25 μg mL^–1^ gentamicin). Grown overnight at 28°C under continuous agitation (200 rpm), *A. tumefaciens* cells were collected until OD_600_ = 0.8–1.2 by centrifugation and resuspended in the infiltration buffer (10 mM 2-(N-morpholino) ethanesulfonic acid [MES], 10 mM MgCl_2_, 200 μM AS). The density of *A. tumefaciens* cells suspension was set to OD_600_ = 0.6–0.8. Next, the *A. tumefaciens* cells containing *pTRV1*, *pTRV2*, or its derivatives, were mixed in a 1:1 ratio and these *A. tumefaciens* suspensions were incubated at room temperature for 3 h. Finally, the *A. tumefaciens* suspension was infiltrated fully on the underside of cotton cotyledons.

### *V. dahliae* Culturing and Infection

*Verticillium dahliae* culturing and infection was performed as described (Zhao et al., 2017) with minor modifications. *V. dahliae* strain Vd991 was streak cultivated onto fresh PDA plates from glycerol stocks and incubated for 1 week at 25°C. The mycelia of one plate (90 mm) was transferred by a sterilized blade into a 200 mL Czapek-Dox medium and cultivated for 1 week with shaking at 200 rpm at 25°C in the dark to collect conidia. After filtrating the fungal cultures with 4 layers of sterilized gauze to remove mycelia, the conidial suspension was collected. The concentration of conidia in suspension was measured using a hemocytometer and was adjusted to approximately 1 × 10^7^ conidia mL^–1^ for infection.

After two weeks of *A. tumefaciens* infiltration, cotton seedlings were inoculated with root-dipping into *V. dahliae* suspension, and then replanted into pots. After growing for another two weeks, the third true leaves of each plant were removed for staining experiments. Pretreated leaves were treated in 10 μM diphenyleneiodonium chloride (DPI, NADPH oxidase inhibitor) for 3 h before ROS staining or trypan blue staining.

### Evaluation of Resistance to Verticillium Wilt

Verticillium wilt disease severity was performed as described (Meng et al., 2018) with minor modifications. The disease index of cotton plants was assessed 21 days and 25 days post-inoculation (dpi) with Vd991. The grade of disease severity was recorded and the disease index (DI) was calculated. The verticillium wilt disease severity level was recorded as five grades: Grade 0 (normal); Grade 1 (disease leaves less than 25%); Grade 2 (disease leaves between 25 and 50%); Grade 3 (disease leaves between 50 and 75%); and Grade 4 (disease leaves over 75%, almost all the dead plants). Disease index was scored with at least 30 plants per treatment and each treatment was repeated at least three times. Data were analyzed by Student’s *t*-test: ^∗^, *p* < 0.05; ^∗∗^, *p* < 0.01.

The lesion area of the transverse section of the cotton stem at 25 dpi with Vd991 was photographed and the browning degree was calculated using imageJ as described (Long et al., 2020). Fungal recovery was performed as described (Zhao et al., 2017).

### Transient Overexpression of *GbRboh5/18* in *G*. *barbadense*

Transient overexpression was performed as described (Li et al., 2018) with minor modifications. Overnight culturing of *A. tumefaciens* LBA4404 cells containing the *pK7WG2.0-GbRboh5/18* plasmid at 28°C until the OD_600_ = 1.2–1.5 *A. tumefaciens* cells were collected and suspended in the infection solution [1/2 MS liquid medium,165 μM acetosyringone (AS), 0.01% Tween-20], with this mixture adjusted to OD_600_ = 0.8–1.0 to infect the cotton seedlings. After seeds of *G. barbadense* L. “Xinhai15” germinated, they were cultured in Hoagland’s solution until their cotyledons had fully expanded and the first true leaf emerged. These seedlings were washed with sterile water and 70% alcohol, and then sterilized in 0.01% HgCl_2_ for 2 min. Seedlings were washed for 2 min with sterile water, 5 times, then placed in a sterile jar to which the infection buffer was added, without Tween-20, for 2 h. After vacuuming for 15 min, the buffer was discarded. The seedlings were incubated in the *A. tumefaciens* suspensions at 28°C for 5 h (at 150 rpm) after seedlings were washed several times with sterile water to reduce the risk of subsequent bacterial contamination. Finally, the transformed seedlings were cultured in 1/2 MS solid medium and grown for 5 days; these seedlings were used to detect the expression levels or for H_2_DCFDA staining.

### ROS Staining, H_2_DCFDA Staining and Trypan Blue Staining

The H_2_O_2_ content and cell death were detected by DAB staining and trypan blue staining, respectively. DAB staining and trypan blue staining were performed as described (Kumar et al., 2014) with minor modifications. Treated or control cotton true leaves were inoculated in 1 mg mL^–1^ of DAB staining solution in 10 mM Tris-acetic acid (pH 5.0) or in 0.4% (W/V) trypan blue staining buffer, and then vacuumed for 10 min. Tissue staining proceeded overnight (DAB staining) or for 3 h (trypan blue staining) at room temperature. Then the staining solution was drained off and absolute ethanol was added before tissue immersion in a boiling water-bath for 10 min to remove chlorophyll. The H_2_O_2_ was visualized as a reddish-brown color, while cell death appeared in blue. The H_2_DCFDA staining was used to detect ROS accumulation in cells. Overexpression cotton true leaves (vector as the negative control) were inoculated in 10 μM H_2_DCFDA solution in PBS buffer (pH 7.5), for 30 min at 37°C, after which the staining solution was drained off and the leaves washed four times with PBS buffer (pH 7.5). Fluorescence was visualized under a Zeiss LSM710 microscope with an excitation light of 488nm.

## Results

### Identification of *Rbohs* in Five *Gossypium* Species

To identify all the *Rboh* genes in the five sequenced *Gossypium* species, we performed a BLASTP search in the diploids *G. arboreum* (A_2_), *G. raimondii* (D_5_), and *G. australe* (G), as well as the tetraploids *G. hirsutum* (AD_1_) and *G. barbadense* (AD_2_). The protein database was queried by PF08022 and PF09068, which are conserved in the AtRbohs. Overall, we found 87 *Rbohs* on the cotton genomes: 13 *Rbohs* in A_2_, 13 *Rbohs* in D_5_, 10 *Rbohs* in G, 24 *Rbohs* in AD_1_, and 27 *Rbohs* in AD_2_ (Table 1). The number of *Rboh* genes in the A_t_ sub-genome of tetraploid *Gossypium* species was the same as that in the diploid *G. arboretum* (A_2_), while the *Rboh* genes in the D_t_ sub-genome of tetraploid *Gossypium* species was less than that in the diploid *G. raimondii* (D5), leaving the remainder 2 *GbRbohs* anchored to unmapped scaffolds. The genome harboring the fewest *Rboh* genes was that of *G. australe* (G).

**TABLE 1:** The number of *Rboh* genes in the five *Gossypium* species.

	*G. arboreum*	*G. raimondii*	*G. australe*	*G. hirsutum* (AD_1_)			*G. barbadense* (AD_2_)			
										Total
	(A_2_)	(D_5_)	(G)	At	Dt	Sca	At	Dt	Sca	
*Rbohs*	13	13	10	13	11	0	13	12	2	87

*“Sca” indicates assembled scaffold. The “At,” “Dt” denote, respectively, the A sub-genomes and D sub-genomes in the tetraploid; “t” indicates tetraploid.*

According to the chromosomal locations of the corresponding genes, we renamed all *Rbohs* consecutively, as *GaRboh1*–*GaRboh13*, *GrRboh1*–*GrRboh13*, *GauRboh1*–*GauRboh10*, *GhRboh1*–*GhRboh24*, and *GbRboh1*–*GbRboh27*. For the 87 *Rboh* genes found in cotton, their encoded proteins consisted of 743–1015 amino acids. The predicted molecular weight of the Rboh proteins in the five *Gossypium* species ranged from 84.2 to 115.47 kDa, with a theoretical *p*I between 8.65 and 9.35. Their predicted subcellular localization showed that 71 of the 87 Rbohs were located in the cytoplasm, except for GaRboh9, GrRboh5, GhRboh2/15, and GbRboh2/15 that were restricted to mitochondria (Supplementary Table S1).

### Phylogenetic Analysis of Rbohs in Five *Gossypium* Species

**FIGURE 1:**
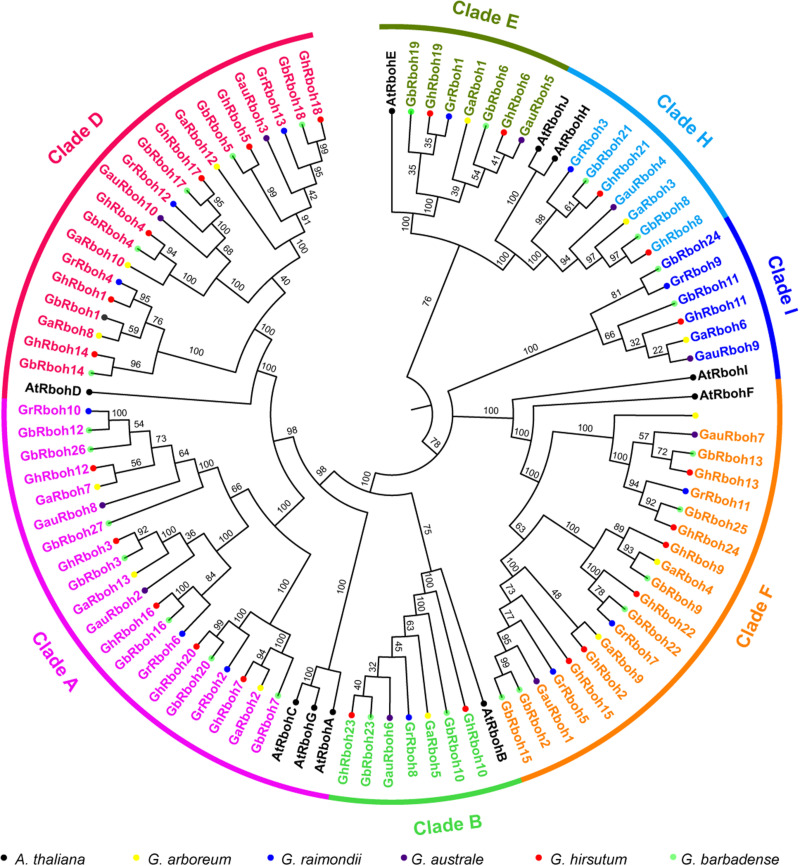
Phylogenetic analysis of Rbohs in cotton and Arabidopsis. A phylogenetic tree of Rboh proteins from *G. arboreum*, *G. raimondii*, *G. australe*, *G. hirsutum*, *G. barbadense*, and *A*. *thaliana*. The full-length amino acid sequences of the Rboh proteins were aligned using ClustalX in MEGA7.0. The unrooted tree was generated by the neighbor-joining (NJ) method (*n* = 1000 bootstraps). Clades are distinguished by different colors. Cotton Rbohs are colored clade specifically and the Arabidopsis Rbohs are in black. Different-colored solid circles indicate genes in different *Gossypium* species or Arabidopsis.

**FIGURE 2:**
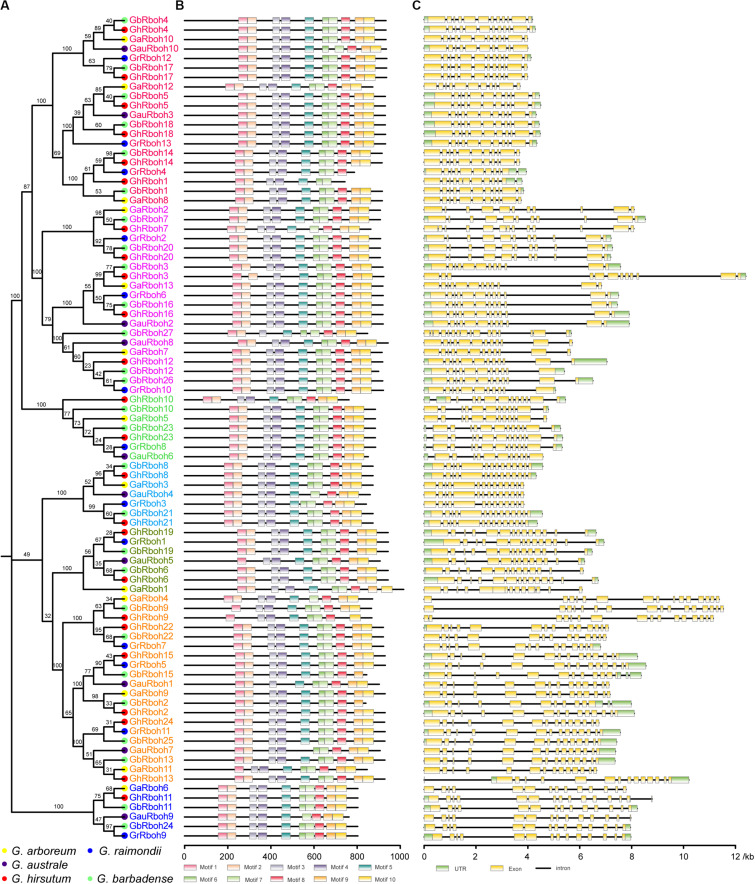
Characterization of the 87 Rbohs in five *Gossypium* species. The characteristics include the homologous relationship, conserved domain location, and exon-intron structure. **(A)** Phylogenetic relationships among the Rbohs in five *Gossypium* species. Rbohs in same clade are indicated by the same color. Different-colored solid circles indicate genes in different *Gossypium* species. **(B)** Conserved domain architecture of cotton Rboh proteins. Motifs were identified through a MEME analysis (http://meme-suite.org/) and protein length was estimated via the scale at the bottom. **(C)** Exon-intron organization analyses of cotton *Rboh* genes. The gene length was estimated by the scale at the bottom through gene structure analyses (GSDS: http://gsds.cbi.pku.edu.cn/). Green boxes indicate 5′- or 3′-untranslated regions. Exons and introns are distinguished in yellow boxes and in black lines, respectively.

To understand their evolutionary relationships, a phylogenetic analysis of the putative 87 Rbohs from the five *Gossypium* species and 10 AtRbohs was carried out. Based on the homologous relationship in Arabidopsis, the 87 Rbohs in cotton were divided into seven clades (named CladeA, -B, -D, -E, -F, -H, and -I according to their homologs in Arabidopsis) (Figures 1, 2A). There were 20 members in CladeA, -D, and -F, 7 members in CladeB, -E, and -H, 6 members in CladeI, respectively. The cotton Rboh members within each clade displayed a high similarity to their orthologs in Arabidopsis. These results suggested the Rbohs in the five *Gossypium* species are highly conserved.

The conserved motif analysis revealed that all these Rboh proteins contained at least 10 conserved motifs: two EF-hands in the N-terminus, six TM domains, and a NADPH-binding motif and a FAD-binding motif in the C-terminal region (Figure 2B). The number and arrangement of conserved domains within the same clade were similar. Comparing their gene structures, the exon-intron organizations revealed that *Rboh* genes in both CladeD and CladeH possessed densely arranged exons, with shorter introns, whereas longer introns characterized the rest of the cotton *Rboh* genes (Figure 2C). These results suggested a similar gene structure and conserved motif arrangement within the same clade.

### Localization of *Rboh* Genes on Five *Gossypium* Chromosomes

**FIGURE 3:**
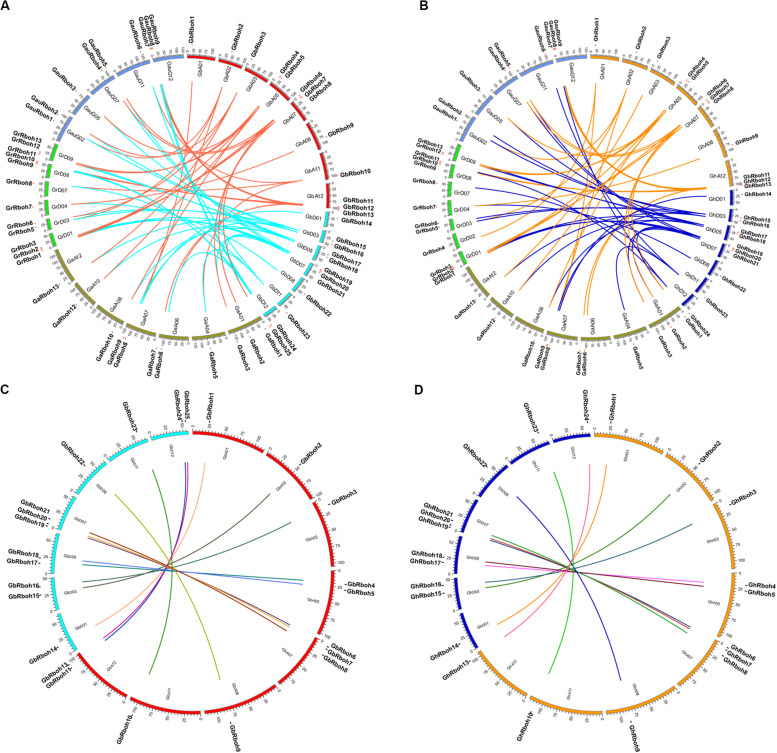
Localization of cotton *Rbohs* on chromosomes. The genomic (At and Dt) analysis of Rboh genes in panels **(A)** *G. barbadense* and **(B)** *G. hirsutum* vis-à-vis the diploid *Gossypium* spp. Different-colored links indicate translocation from the A or D sub-genome of an allotetraploid cotton to the diploid cotton. Lined gene coupling represents a shared identity between them that exceeded 91%. The inter-genomic (At and Dt) analyses of *Rboh* genes in panels **(C)** *G. barbadense* and **(D)** *G. hirsutum* between the A and D sub-genomes. Different-colored lines indicate gene couples whose identity is over 98%, depicted using the Circos genome visualization tool (http://www.circos.ca/).

To better understand their evolutionary relationships, we further analyzed the orthologous and paralogous *Rboh* genes in the five *Gossypium* species. Many gene blocks were found among the chromosomes of two sub-genomes of *G. hirsutum* and *G. barbadense*, marked by similarities to those in the sequenced diploid cotton genomes (A_2_, D_5_, and G). Compared to *G. raimondii*, *G. arboreum*, and *G. australe*, there, respectively were 22, 26, and 18 pairs of putative outparalogous genes in *G. barbadense* (Figure 3A). In *G. hirsutum*, there were 18, 20, and 14 pairs of putative orthologous *Rbohs* genes when compared to *G. raimondii*, *G. arboretum*, and *G. australe*, respectively (Figure 3B).

Further analysis of the paralogous Rboh genes, between the A and D subgenomes in the two tetraploid cotton species, showed that 24 of 27 could be one-to-one paired in *G. barbadense* between the A and D sub-genomes, in addition to *GbRboh12*, *GbRboh26*, and *GbRboh27* (Figure 3C). There were 11 syntenic gene blocks in *G. hirsutum*, with the exception of *GhRboh11* and *GhRboh12* (Figure 3D); these paired genes may exist as alleles on chromosomes between the A and D sub-genomes. These results implied that Rbohs are generally highly conserved between A and D sub-genomes during the evolution of tetraploid cotton plants.

### CladeD (mainly *GbRboh5/18*) Response to Pathogen and Hormones in *G*. *barbadense*

**FIGURE 4:**
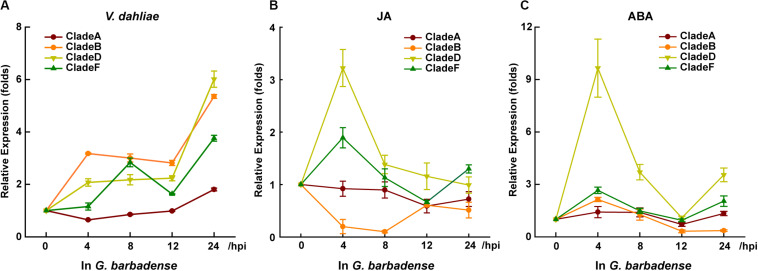
*GbRbohs* in the CladeD respond to *V. dahliae* infection and hormones in G. *barbadense*. Quantitative analyses of each clade *GbRbohs*’ expression levels in “Xinhai15” cotton plant leaves treated with **(A)** *V*. *dahliae*, **(B)** jasmonic acid, and **(C)** abscisic acid. Total RNAs were extracted from leaves of 3-week-old seedlings at 0, 4, 8, 12, and 24 h post-inoculation. The values are the mean ± SD for three technical replicates. Relative expression levels of all clade genes were determined after normalizing to the expression level at 0 h, which was set to 1.0. hpi, hours post-inoculation. The experiments were repeated at least three times, with similar results.

To detect which Rbohs may contribute to plant resistance against verticillium wilt, or which can respond to hormones in *G*. *barbadense*, we designed specific primers for the conserved regions to detect the post-treatment level of transcripts for each clade’s genes. Within 24 h of treatment with *V. dahliae*, JA, or ABA, the transcripts of CladeA, -B, -D, and -F showed dramatic or slight changes, whereas the transcripts of *GbRbohs* in the other remaining three clades went undetected (Figure 4). Among the four responsive clades, CladeD underwent the starkest fold-change during the treatment with *V. dahliae* (6-fold at 24 h), JA (3.2-fold at 4 h), and ABA (9.6-fold at 4 h).

**FIGURE 5:**
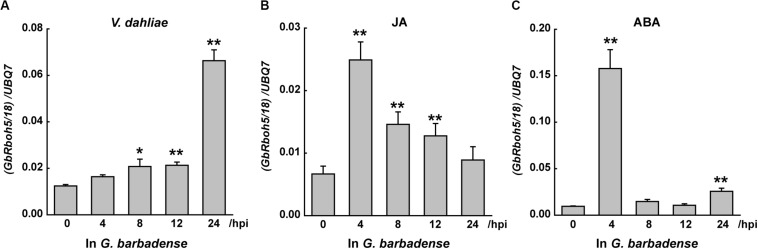
*GbRboh5/18* are the main functional genes of CladeD in *G*. *barbadense*. Quantitative analyses of *GbRboh5/18* expression levels in “Xinhai15” cotton plant leaves inoculated with **(A)** *V*. *dahliae*, **(B)** jasmonic acid, and **(C)** abscisic acid. Total RNAs were extracted from leaves of 3-week-old seedlings at 0, 4, 8, 12, and 24 h post-inoculation. The experiments were repeated three times, with similar results. The values are the means ± SD for three technical replicates. Transcript levels of each gene couple were first normalized to *UBQ7*. Asterisks indicate significant differences compared with 0 h under same treatment (*p < 0.05; **p < 0.01, Student’s *t*-test).

In *G*. *barbadense*, CladeD included three gene couples: *GbRboh1/14*, *GbRboh4/17*, and *GbRboh5/18*. We further detected their respective transcript levels by Q-PCR to identify which members contributed chiefly. The transcript of *GbRboh5/18* was clearly induced by *V. dahliae*, or the JA, or ABA treatments (Figure 5), whereas the transcripts of *GbRboh1/14* and *GbRboh4/17* were not detected. Furthermore, the trend in the variation of *GbRboh5/18*’s transcript level was consistent with CladeD; hence we deduced that *GbRboh5/18* is the cardinal member of CladeD in *G*. *barbadense*.

### Knock-Down of *GbRboh5/18* Attenuates Resistance to *V. dahliae* in *G. barbadense*

**FIGURE 6:**
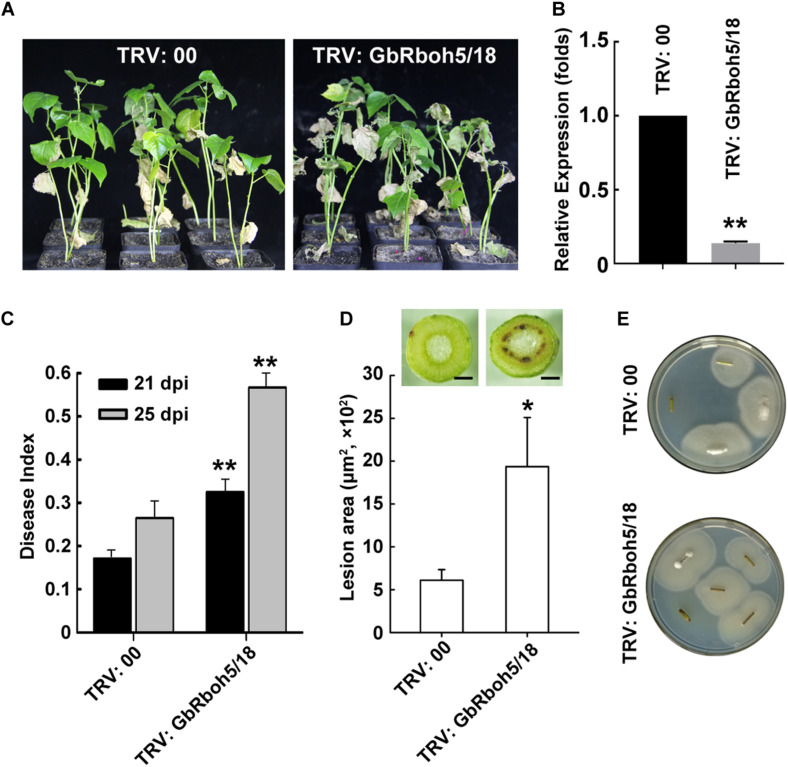
Knock-down of *GbRboh5/18* attenuates plant resistance to *V. dahliae*. **(A)** Disease phenotype of cotton plants (“Xinhai15”) at 25 days post-infection with *V*. *dahliae* strain Vd991. Seedlings were inoculated with *V. dahliae* two weeks after VIGS and photographed 25 days later. **(B)** Expression of *GbRboh5/18* in *GbRboh5/18-VIGS* and control plants. *UBQ7* was the internal reference gene (**p < 0.01; Student’s *t*-tests). **(C)** Disease index of *GbRboh5/18-VIGS* and control plants at 21 and 25 dpi with *V. dahliae*. Asterisks indicate significant differences compared with TRV: 00 at same time (*p < 0.05; **p < 0.01, Student’s *t*-test). **(D)** Lesion area of *GbRboh5/18-VIGS* and control plants’ stems at 25 dpi with *V. dahliae*. Transverse section of stems were photographed with an OLYMPUS CX31 microscope. The values are the mean ± SD, *n* ≥ 30. Bar = 0.5 mm. **(E)** Fungal recovery assay. Sterile stems approximately 1 cm from same position of plants was plated on PDA plates at 25°C to allow *V. dahliae* recovery; photographs were taken 3 days post-inoculation. All experiments were repeated at least three times, with similar results.

To verify the function of GbRbohD in cotton plant resistance to *V. dahliae*, we utilized VIGS, a powerful technique, to achieve knock-down in the transcript activity of *GbRboh5/18*, the gene mainly responsible in CladeD. When “Xinhai15” (cultivar of *G. barbadense*) seedlings had grown for 10 days, we infiltrated cotyledons with TRV: 00 (as negative control) or TRV: GbRboh5/18. After 2 weeks of treatment with *V. dahliae*, the *GbRboh5/18-VIGS* plants displayed more severe disease symptoms than the control plants, such as wilted leaves and dark vascular bundles (Figure 6A). The successful knock-down of the *GbRboh5/18* transcript was confirmed by the Q-PCR analysis (Figure 6B), and the disease index was consistent with the plant phenotype (Figure 6C). Further, a lesion area analysis and fungal recovery assay closely resembled the phenotype and disease index (Figures 6D,E). These results suggested that the knock-down of *GbRboh5/18* transcription is able to attenuate plant resistance to *V. dahliae* infection.

### GbRboh5/18 Promotes ROS Accumulation in *G. barbadense*

**FIGURE 7:**
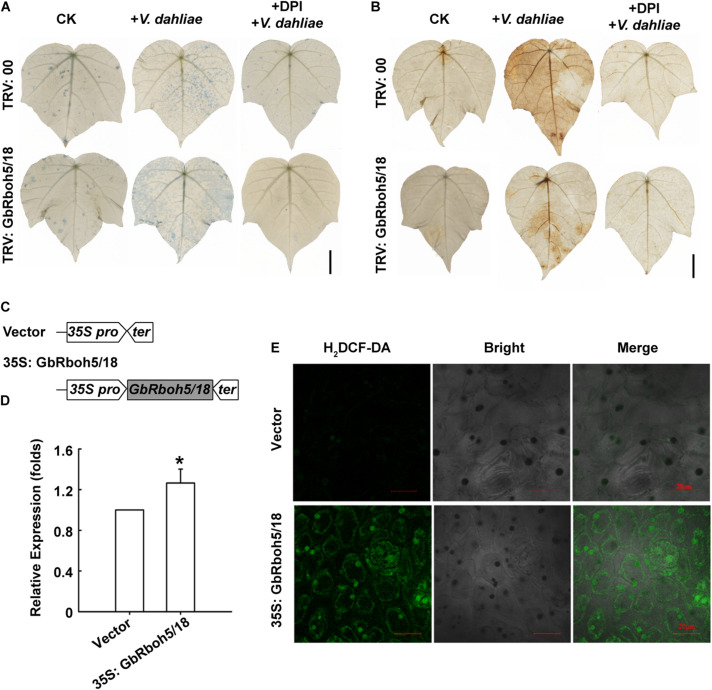
GbRboh5/18 promotes ROS accumulation in *G. barbadense*. **(A)** Trypan blue staining of cell death and **(B)** DAB staining of ROS in *GbRboh5/18-VIGS* and control leaves. Leaves were stained at 48 h post-inoculation with or without *V. dahliae*. Leaves were pretreated with or without 10 μM DPI before trypan blue staining or DAB staining. Bar = 1 cm. **(C)** Schematic diagram showing the structure of overexpression vector. Gray-filled squares indicate ORF of *GbRboh5/18*; the left pentagon and right pentagon are the 35S promoter and 35S terminator, respectively. **(D)** Expression of *GbRboh5/18* in *GbRboh5/18-OE* and vector plant leaves. *UBQ7* was the internal reference gene. (*p < 0.05; Student’s *t*-tests). **(E)** H_2_DCF-DA fluorescence probe of ROS in *GbRboh5/18-OE* and vector leaves cells. Bar = 20 μm. After 5 days of growth, the first true leaves were used to Q-PCR or H_2_DCF-DA staining. All experiments were repeated at least three times with similar results.

To clarify the function of GbRboh5/18, we detected cell death in the *GbRboh5/18-VIGS* plants and control plants, via trypan blue staining. There was no significant difference between *GbRboh5/18-VIGS* and control leaves, but more of the blue stain appeared in *GbRboh5/18-VIGS* leaves than control leaves after infection with *V. dahliae*. Pretreatment with DPI, an inhibitor of the NADPH oxidase, before *V. dahliae* infection, resulted in almost no staining in all leaves (Figure 7A). Further, the DAB staining showed less ROS accumulation in *GbRboh5/18-VIGS* leaves than in control leaves after *V. dahliae* infection, yet this did not occur in both groups leaves when they were pretreated with DPI (Figure 7B). These results indicated that the knock-down of *GbRboh5/18* caused more cell death mediated by less ROS accumulation when plants were infected with the *V. dahliae* pathogen.

Nonetheless, we constructed the *GbRboh5/18*-OE vector to verify the functional mechanism of *GbRboh5/18* (Figure 7C). We utilized a transient overexpression strategy to detect ROS accumulation in cotton seedlings. Overexpression of *GbRboh5/18* was confirmed by the Q-PCR analysis (Figure 7D). The H_2_DCF-DA fluorescent assay was used to observe ROS accumulation in *GbRboh5/18-OE* leaves, which revealed stronger fluorescent signals in *GbRboh5/18*-OE than in the control (Figure 7E). These results showed that GbRboh5/18 can increase the ROS accumulation.

## Discussion

Rbohs, a group of plant-specific NADPH oxidases, have been phylogenetically and functionally characterized for a variety of plant species (Torres et al., 1998; Xia et al., 2009; Kaur and Pati, 2018), but not yet clearly for cotton (*Gossypium* spp.). Five *Gossypium* genomes were sequenced and assembled over the past few years (Li et al., 2014a, 2015; Zhang et al., 2015; Du et al., 2018; Ma et al., 2018, 2019; Cai et al., 2019; Hu et al., 2019; Wang et al., 2019). With the development of sequencing and assembly technology, information of genomes is becoming more and more complete. Utilizing bioinformatics, Q-PCR, VIGS, and plant-pathogen biology, in this study we systematically reported on the *Rboh* gene family in five sequenced *Gossypium* species. This integrated approach included building a phylogenetic tree of the *Rboh* genes in cotton and Arabidopsis; analyses of gene structure and conserved domains, and transcript levels’ response to *V. dahliae*; and verifying the function of *GbRboh5/18* in conferring resistance to *V. dahliae*. Collectively, this provides a basic understanding of cotton *Rboh* genes and highlights the key role of *GbRboh5/18* in responses to *V. dahliae*.

We systematically identified 87 *Rboh* genes in these representative five sequenced *Gossypium* species: *G. arboretum*, *G. raimondii*, *G. australe*, *G. hirsutum*, and *G. barbadense*. Both *G. arboretum* and *G. raimondii* harbored 13 Rboh genes, a result that agrees with the findings of Zhang et al. (2020). The main difference with that work is that we found 24 and 27 *Rboh* genes in *G. hirsutum* and *G. barbadense*, while they found 26 and 19, respectively (Wang et al., 2020). For *G. hirsutum*, 4 of 26 *GhRbohs* were anchored to unmapped scaffolds in the NAU-NBI v1.1 assembly genome (assembled in 2015), whereas we found that all 24 *GhRbohs* in the HAU v1.0 assembly genome (assembled in 2018) mapped onto the A_t_ or D_t_ sub-genome chromosomes. For *G. barbadense*, we found more *GbRbohs* in the HAU v2.0 assembly genome (assembled in 2018) than in the NAU assembly genome (Table 1, Supplementary Table S1). This disparity may be due to recent advances in assembly technology that have improved the quantity and quality of genomic information, resulted in a more accurate genome. In addition, this study uncovered 10 *GauRbohs* in the *G. austral* genome, making it now possible to further explore the function of the *Rboh* gene family in this species.

The number of *Rboh* genes in *G. hirsutum* was less than its sum in the two diploids, suggesting that gene loss events happened in the *Rboh* gene family during the polyploidization of *G. hirsutum*. By contrast, the number of *Rboh* genes in *G. barbadense* was more than the sum of the *Rboh* genes’ number in the A_2_ and D_5_ genomes, suggesting gene expansion occurred in the *Rboh* gene family during the polyploidization of *G. barbadense*. These results are consistent with previous work showing that many gene loss and duplication events have occurred in allotetraploid cotton plants (Li et al., 2015; Zhang et al., 2015; Wang et al., 2019; Yang et al., 2019). According to their phylogenetic relationships with Arabidopsis orthologs, the Rbohs were assigned to seven clades. In addition to CladeA and -I, there were twice as many genes of other clades in the tetraploids as in the diploid ancestors (*G. arboretum* and *G. raimondii*). This suggests that the *Rboh* gene family was conserved during the evolution of cotton.

Rbohs play pivotal roles in ROS production during the PTI and ETI responses in Arabidopsis and rice (Pogany et al., 2009; Chaouch et al., 2012; Dubiella et al., 2013). Verticillium wilt, a “cancer” of cotton production, is a vascular bundle disease caused by the fungus *V*. *dahliae* (Fradin et al., 2009). Overexpression of polyamine oxidase GhPAO contributes to the resistance of plants against *V. dahliae* by elevating the level of H_2_O_2_ accumulation (Mo et al., 2015). These previous findings contended that ROS content influences plant resistance to *V. dahliae*. Yet in our study we did not detect the transcripts of *CladeE* (*GbRboh6/19*), *CladeH* (*GbRboh8/21*), or *CladeI* (*GbRboh11/24*) when plants were treated with either the pathogen or hormones. Accordingly, these results lead us to speculate that *CladeE*, *CladeH*, and *CladeI* may participate in certain physiological developmental processes, but not in the cotton plant defense response against this pathogen.

AtRbohD maintains hydrogen peroxide (H_2_O_2_) homeostasis in Arabidopsis plants under different stresses (Kobayashi et al., 2012; Morales et al., 2016; Yao et al., 2017). The latest findings suggest that RbohD-dependent H_2_O_2_ production is regulated by the PTP-MPK3/6-WRKY pathway in the response against Vd-toxins in Arabidopsis (Zhao et al., 2020). Since *GbRboh5/18* mainly contributes to *CladeD*, we also determined the function of *GbRboh5/18*, via VIGS and transient overexpression experiments. Knock-down of *GbRboh5/18* resulted in a lowered ROS content and reduced resistance to *V. dahliae*, whereas overexpressing *GbRboh5/18* resulted in higher ROS accumulations (Figures 6, 7). So we may conclude that *GbRboh5/18* enhances cotton plant resistance by increasing its ROS accumulation.

In conclusion, we showed that the *Rboh* gene family and *GbRboh5/18*, in particular, contributed to resistance against verticillium wilt in cotton. The molecular functional mechanism of *GbRboh5/18* in the regulation of H_2_O_2_ production now awaits investigation.

## Data Availability Statement

Publicly available datasets were analyzed in this study. This data can be found here: https://www.cottongen.org/ [BGI-CGB v2.0 assembly genome for *G. arboreum* (A2)], [JGI v2.0 assembly data for *G. raimondii* (D5)], [HAU v1.0 assembly genome for *G. hirsutum* (AD1)], [HAU v2.0 assembly genome for *G. barbadense* (AD2)]; https://www.ncbi.nlm.nih.gov/ [assembly CCRI_HENU_Gaus_1.1 for *G. australe* (G)].

## Author Contributions

YC and BL designed the experiments. BL and SG analyzed the genes’ data. QS and RG performed the Q-PCR, VIGS, and the ROS detection. BL and YZ carried out the overexpression of cotton seedlings. JL constructed the phylogenetic tree. YC and YFC wrote the manuscript. All authors read and approved the final manuscript.

## Conflict of Interest

The authors declare that the research was conducted in the absence of any commercial or financial relationships that could be construed as a potential conflict of interest.

## Footnotes

1 http://pfam.xfam.org/

2 https://www.cottongen.org/

3 https://www.ncbi.nlm.nih.gov/

4 http://web.expasy.org/protparam

5 https://abi-services.informatik.uni-tuebingen.de/sherloc2/webloc.cgi

6 http://www.arabidopsis.org

7 https://www.megasoftware.net

8 http://gsds.cbi.pku.edu.cn/

9 http://meme-suite.org/tools/meme

10 http://www.circos.ca/
