# Supplementary material for: Comprehensive Analysis of Respiratory Burst Oxidase Homologs (Rboh) Gene Family and Function of GbRboh5/18 on Verticillium Wilt Resistance in Gossypium barbadense
Source: Front Genet. 2020 Sep 11;11:788. doi: 10.3389/fgene.2020.00788 (PMC7517705; doi:10.3389/fgene.2020.00788)
Supplement: Supplementary file 1 [file Table_1.DOCX]

**Table S1: Genome-wide identification and analysis of *Rboh* genes in five sequenced *Gossypium* species**

| Gene Name | Gene ID | Chromosome Location | Ori | CDS (bp) | Protein (AA) | MW (kDa) | *p*I | Subcellular Localization |
| --- | --- | --- | --- | --- | --- | --- | --- | --- |
| GaRboh1 | Cotton_A_05673 | ChrA01: 2282778-2288775 | - | 3045 | 1015 | 115.47 | 9.10 | Cyt |
| GaRboh2 | Cotton_A_03631 | ChrA01: 66996228-67004202 | + | 2724 | 907 | 102.94 | 9.23 | Cyt |
| GaRboh3 | Cotton_A_24667 | ChrA01: 119438005-119441783 | + | 2622 | 873 | 99.90 | 9.13 | Cyt |
| GaRboh4 | Cotton_A_31123 | ChrA03: 11466232-11477440 | - | 2616 | 871 | 99.85 | 9.09 | Cyt |
| GaRboh5 | Cotton_A_29320 | ChrA04: 62602318-62606964 | - | 2655 | 884 | 100.82 | 9.19 | Cyt |
| GaRboh6 | Cotton_A_13344 | ChrA06: 98939017-98946687 | + | 2412 | 803 | 91.05 | 9.08 | Cyt |
| GaRboh7 | Cotton_A_07974 | ChrA06: 115992732-115998284 | - | 2748 | 915 | 103.64 | 9.17 | Cyt |
| GaRboh8 | Cotton_A_33534 | ChrA07: 104586422-104590103 | - | 2751 | 916 | 104.13 | 9.13 | Cyt |
| GaRboh9 | Cotton_A_38536 | ChrA07: 111881839-111888901 | + | 2790 | 929 | 105.97 | 9.28 | Mit |
| GaRboh10 | Cotton_A_13752 | ChrA08: 15573735-15577658 | - | 2799 | 933 | 105.27 | 9.04 | Cyt |
| GaRboh11 | Cotton_A_23171 | ChrA10: 30973779-30980330 | + | 2541 | 846 | 95.66 | 9.12 | Cyt |
| GaRboh12 | Cotton_A_18772 | ChrA10: 46503475-46507115 | + | 2619 | 872 | 98.56 | 9.12 | Cyt |
| GaRboh13 | Cotton_A_16216 | ChrA12: 17846186-17852922 | + | 2760 | 919 | 103.99 | 8.82 | Cyt |
| GrRboh1 | Gorai.001G017400 | ChrD01: 1596440-1603268 | - | 2832 | 944 | 106.98 | 8.87 | Cyt |
| GrRboh2 | Gorai.001G053300 | ChrD01: 5075477-5082571 | + | 2724 | 908 | 102.74 | 9.07 | Cyt |
| GrRboh3 | Gorai.001G106500 | ChrD01: 11992089-11995872 | - | 2526 | 842 | 95.96 | 9.10 | Cyt |
| GrRboh4 | Gorai.002G128200 | ChrD02: 19755039-19758915 | + | 2361 | 787 | 89.00 | 8.94 | Cyt |
| GrRboh5 | Gorai.003G085100 | ChrD03: 21397172-21405595 | - | 2790 | 930 | 105.88 | 9.30 | Mit |
| GrRboh6 | Gorai.003G117900 | ChrD03: 35711880-35719260 | - | 2760 | 920 | 103.74 | 8.90 | Cyt |
| GrRboh7 | Gorai.004G137300 | ChrD04: 38403501-38410198 | - | 2766 | 922 | 105.06 | 9.32 | Cyt |
| GrRboh8 | Gorai.007G299500 | ChrD07: 51137164-51142401 | + | 2655 | 885 | 100.86 | 9.19 | Cyt |
| GrRboh9 | Gorai.008G199100 | ChrD08: 48380739-48388590 | - | 2409 | 803 | 90.66 | 9.11 | Cyt |
| GrRboh10 | Gorai.008G212100 | ChrD08: 49852054-49858167 | - | 2763 | 921 | 104.43 | 9.02 | Cyt |
| GrRboh11 | Gorai.008G250500 | ChrD08: 53444134-53451583 | + | 2787 | 929 | 105.77 | 9.35 | Cyt |
| GrRboh12 | Gorai.009G202500 | ChrD09: 15702162-15706217 | + | 2811 | 937 | 105.41 | 9.03 | Cyt |
| GrRboh13 | Gorai.009G273400 | ChrD09: 22885462-22889737 | + | 2793 | 931 | 104.66 | 9.13 | Cyt |
| GauRboh1 | GAUG00043055 | Chr02：67983122-67990146 | + | 2706 | 902 | 102.68 | 9.33 | Cyt |
| GauRboh2 | GAUG00042412 | Chr02：96798616-96806407 | - | 2760 | 920 | 103.96 | 8.92 | Cyt |
| GauRboh3 | GAUG00046305 | Chr05：82390154-82394402 | - | 2790 | 930 | 104.49 | 9.17 | Cyt |
| GauRboh4 | GAUG00049484 | Chr07：94854473-94858254 | + | 2580 | 859 | 98.19 | 9.15 | Cyt |
| GauRboh5 | GAUG00035493 | Chr07：113668379-113674470 | + | 2718 | 906 | 102.65 | 9.20 | Cyt |
| GauRboh6 | GAUG00005662 | Chr11：128420535-128425045 | + | 2553 | 851 | 96.97 | 9.16 | Cyt |
| GauRboh7 | GAUG00013685 | Chr12：4959781-4967051 | - | 2718 | 906 | 102.75 | 9.26 | Cyt |
| GauRboh8 | GAUG00014079 | Chr12：11118724-11124350 | + | 2828 | 943 | 106.84 | 9.19 | Cyt |
| GauRboh9 | GAUG00014178 | Chr12：13712963-13720816 | + | 2286 | 762 | 85.95 | 9.18 | Cyt |
| GauRboh10 | GAUG00003599 | Chr13：28148443-28152378 | - | 2805 | 935 | 105.15 | 9.01 | Cyt |
| GhRboh1 | Ghir_A01G010350 | ChrA01: 24742926-24746639 | + | 2232 | 743 | 84.20 | 9.03 | Cyt |
| GhRboh2 | Ghir_A02G011270 | ChrA02: 50025572-50033556 | - | 2790 | 929 | 105.96 | 9.28 | Mit |
| GhRboh3 | Ghir_A03G006520 | ChrA03: 12892217-12904437 | + | 2772 | 923 | 104.86 | 8.65 | Cyt |
| GhRboh4 | Ghir_A05G019830 | ChrA05: 18988862-18993079 | + | 2802 | 933 | 105.37 | 9.11 | Cyt |
| GhRboh5 | Ghir_A05G026340 | ChrA05: 27533828-27538252 | + | 2793 | 930 | 104.57 | 9.13 | Cyt |
| GhRboh6 | Ghir_A07G001750 | ChrA07: 1840014-1846621 | - | 2832 | 943 | 106.96 | 8.87 | Cyt |
| GhRboh7 | Ghir_A07G005180 | ChrA07: 5890378-5898342 | + | 2592 | 863 | 98.30 | 9.19 | Cyt |
| GhRboh8 | Ghir_A07G010230 | ChrA07: 15880871-15885121 | - | 2622 | 873 | 99.94 | 9.13 | Cyt |
| GhRboh9 | Ghir_A08G012130 | ChrA08: 86293049-86304035 | - | 2607 | 868 | 99.54 | 9.34 | Cyt |
| GhRboh10 | Ghir_A11G028920 | ChrA11: 111568762-111574127 | + | 2289 | 762 | 87.38 | 9.20 | Cyt |
| GhRboh11 | Ghir_A12G019370 | ChrA12: 97368301-97376964 | - | 2412 | 803 | 91.07 | 9.11 | Cyt |
| GhRboh12 | Ghir_A12G020750 | ChrA12: 99228913-99235944 | - | 2757 | 918 | 104.07 | 9.16 | Cyt |
| GhRboh13 | Ghir_A12G024550 | ChrA12: 103446685-103456745 | + | 2787 | 928 | 105.74 | 9.29 | Cyt |
| GhRboh14 | Ghir_D01G011360 | ChrD01: 19134575-19138198 | + | 2748 | 915 | 104.19 | 8.99 | Cyt |
| GhRboh15 | Ghir_D03G008610 | ChrD03: 30019899-30027999 | + | 2790 | 929 | 105.83 | 9.31 | Mit |
| GhRboh16 | Ghir_D03G012320 | ChrD03: 41737976-41745764 | - | 2760 | 919 | 103.87 | 8.82 | Cyt |
| GhRboh17 | Ghir_D05G019850 | ChrD05: 17286126-17290050 | + | 2811 | 936 | 105.21 | 8.96 | Cyt |
| GhRboh18 | Ghir_D05G026370 | ChrD05: 24976871-24981276 | + | 2796 | 931 | 104.91 | 9.06 | Cyt |
| GhRboh19 | Ghir_D07G001790 | ChrD07: 1799162-1805691 | - | 2832 | 943 | 106.98 | 8.87 | Cyt |
| GhRboh20 | Ghir_D07G005190 | ChrD07: 5411818-5418902 | + | 2724 | 907 | 102.70 | 9.00 | Cyt |
| GhRboh21 | Ghir_D07G010260 | ChrD07: 12574304-12578597 | - | 2622 | 873 | 100.00 | 9.18 | Cyt |
| GhRboh22 | Ghir_D08G012790 | ChrD08: 44059181-44066175 | - | 2766 | 921 | 105.13 | 9.28 | Cyt |
| GhRboh23 | Ghir_D11G029100 | ChrD11: 61716478-61721732 | + | 2655 | 884 | 100.80 | 9.19 | Cyt |
| GhRboh24 | Ghir_D12G024550 | ChrD12: 58645462-58652100 | + | 2784 | 927 | 105.64 | 9.35 | Cyt |
| GbRboh1 | Gbar_A01G010750 | ChrA01: 24286466-24290239 | + | 2751 | 916 | 104.20 | 9.07 | Cyt |
| GbRboh2 | Gbar_A02G011090 | ChrA02: 47543331-47551199 | - | 2523 | 840 | 95.97 | 9.24 | Mit |
| GbRboh3 | Gbar_A03G006550 | ChrA03: 11615851-11623307 | + | 2760 | 919 | 104.07 | 8.77 | Cyt |
| GbRboh4 | Gbar_A05G019180 | ChrA05: 18193951-18198063 | + | 2802 | 933 | 105.34 | 9.11 | Cyt |
| GbRboh5 | Gbar_A05G025370 | ChrA05: 26303635-26308007 | + | 2793 | 930 | 104.50 | 9.13 | Cyt |
| GbRboh6 | Gbar_A07G001790 | ChrA07: 1945648-1951689 | - | 2832 | 943 | 106.99 | 8.92 | Cyt |
| GbRboh7 | Gbar_A07G004990 | ChrA07: 5772281-5780682 | + | 2724 | 907 | 102.89 | 9.20 | Cyt |
| GbRboh8 | Gbar_A07G009940 | ChrA07: 15503402-15507905 | - | 2622 | 873 | 99.91 | 9.13 | Cyt |
| GbRboh9 | Gbar_A08G012480 | ChrA08: 83807061-83818429 | - | 2601 | 866 | 98.50 | 9.21 | Cyt |
| GbRboh10 | Gbar_A11G028500 | ChrA11: 100943001-100948068 | + | 2655 | 884 | 100.78 | 9.22 | Cyt |
| GbRboh11 | Gbar_A12G019480 | ChrA12: 92257421-92265518 | - | 2412 | 803 | 91.04 | 9.08 | Cyt |
| GbRboh12 | Gbar_A12G020850 | ChrA12: 94244938-94250261 | - | 2700 | 899 | 101.91 | 9.16 | Cyt |
| GbRboh13 | Gbar_A12G024320 | ChrA12: 98153263-98160514 | + | 2787 | 928 | 105.68 | 9.29 | Cyt |
| GbRboh14 | Gbar_D01G011310 | ChrD01: 19243764-19247386 | + | 2748 | 915 | 104.23 | 9.07 | Cyt |
| GbRboh15 | Gbar_D03G008080 | ChrD03: 29039411-29047654 | + | 2535 | 844 | 96.08 | 9.33 | Mit |
| GbRboh16 | Gbar_D03G011690 | ChrD03: 40747073-40754408 | - | 2760 | 919 | 103.90 | 8.82 | Cyt |
| GbRboh17 | Gbar_D05G019860 | ChrD05: 17159550-17163458 | + | 2811 | 936 | 105.30 | 8.96 | Cyt |
| GbRboh18 | Gbar_D05G026260 | ChrD05: 24711330-24715695 | + | 2793 | 930 | 104.80 | 9.06 | Cyt |
| GbRboh19 | Gbar_D07G001790 | ChrD07: 1731908-1738286 | - | 2832 | 943 | 106.98 | 8.87 | Cyt |
| GbRboh20 | Gbar_D07G005280 | ChrD07: 5424623-5431771 | + | 2724 | 907 | 102.73 | 9.07 | Cyt |
| GbRboh21 | Gbar_D07G010330 | ChrD07: 12615216-12619700 | - | 2622 | 873 | 99.97 | 9.20 | Cyt |
| GbRboh22 | Gbar_D08G013170 | ChrD08: 41468141-41475052 | - | 2766 | 921 | 105.03 | 9.32 | Cyt |
| GbRboh23 | Gbar_D11G028960 | ChrD11: 58533393-58538570 | + | 2655 | 884 | 100.83 | 9.19 | Cyt |
| GbRboh24 | Gbar_D12G019720 | ChrD12: 50314666-50322501 | - | 2409 | 802 | 90.80 | 9.03 | Cyt |
| GbRboh25 | Gbar_D12G024270 | ChrD12: 55116876-55124188 | + | 2787 | 928 | 105.74 | 9.35 | Cyt |
| GbRboh26 | Gbar_D12G028850 | Scaffold153: 204872-211281 | + | 2757 | 918 | 104.16 | 9.12 | Cyt |
| GbRboh27 | Gbar_Scaffold499G000010 | Scaffold499: 556-6143 | + | 2547 | 848 | 97.10 | 8.93 | Cyt |

Gene Name: gene named based on the corresponding location on chromosome; Ori: orientation, “+” represent forward and “-” represent reverse;

MW: predicted molecular weight; AA: amino acid; *p*I: predicted isoelectric point; Cyt: cytoplasmic; Mit: mitochondria.
