# Supplementary material for: Comprehensive Analysis of Respiratory Burst Oxidase Homologs (Rboh) Gene Family and Function of GbRboh5/18 on Verticillium Wilt Resistance in Gossypium barbadense
Source: Front Genet. 2020 Sep 11;11:788. doi: 10.3389/fgene.2020.00788 (PMC7517705; doi:10.3389/fgene.2020.00788)
Supplement: Supplementary file 2 [file Table_2.DOCX]

**Table S2: Primers in this study**

| Primer Name | Oligonucleotide (5'-3') |
| --- | --- |
| **Primers for Q-PCR** |  |
| CladeA-QF | GGATAAAGATGCAGATGGAAGAAT |
| CladeA-QR | GCATATTCCTCAGCTTGTTTCTG |
| CladeB-QF | GCTCGCAGAAGAGGGATAAC |
| CladeB-QR | CTCAATGTACCCAAGGTTGTC |
| CladeD-QF | GAAAGCTTTGACTCTAGACTCC |
| CladeD-QR | GCATATTCTTCAGCCTGTTTCT |
| CladeE-QF | GAGGAGCTTCATGATTTCTGGTT |
| CladeE-QR | GAGGAGCTTCATGATTTCTGGTT |
| CladeF-QF | CAATGCATGGGAAGAAGTTG |
| CladeF-QR | AAGAAGATCTGGAGGCGAGAAT |
| CladeH-QF | CTGCGTTGGAATGGGAGATT |
| CladeH-QR | GCATATGTTCCGGCTTGATCTT |
| CladeI-QF | GAGGTTGTGAAATGGTCGGAT |
| CladeI-QR | GCGTATTCTTCGGCTTCCTT |
| GbRboh1/14-QF | AATACAACTCAAACGCTTCTGG |
| GbRboh1/14-QR | GCCTGTTTCTGAATAGTAGCAAGT |
| GbRboh4/17-QF | TGCAAACAGACTTTCAAACATTC |
| GbRboh4/17-QR | TTGTGAATCATGATGTATCCAGC |
| GbRboh5/18-QF | GACTTGACCGCCTCTACGAAT |
| GbRboh5/18-QR | CTGACTTCTTCTTCTGTTATTCTTCC |
| GbRboh2/15-QF | TATGGTGGACAAGAATGAAGATGGTAGAATA |
| GbRboh2/15-QR | TGAGGACACAATAATGCATGACTACA |
| GbRboh9/22-QF | CAGGTTATGGGTTACTGTCTCC |
| GbRboh9/22-QR | CACTAGAGGACTTGATAAGCATAGG |
| GbRboh13/25-QF | ACAGAAGAGTGCTTTTAATGTTAT |
| GbRboh13/25-QR | TCTATAGTTATCATTAGAAGAGCTGAT |
| UBQ7-QF | GAAGGCATTCCACCTGACCAAC |
| UBQ7-QR | CTTGACCTTCTTCTTCTTGTGCTTG |
| **Primers for Vector construct** |  |
| GbRboh5/18-F | ATGAGGAACGATGATGGGAGAGGAG |
| GbRboh5/18-R | GCCGTTAAAAATTTTCTTTGTGGAATTCG |
| GbRboh5/18-VIGS-F | CGCGGATCCGAGGAACGATGATGGGAGA |
| GbRboh5/18-VIGS-R | CGGGGTACCCCTTGAACGCTGTGAACC |
| GbRboh5/18-OE-F | GGGGACAAGTTTGTACAAAAAAGCAGGCT ATGAGGAACGATGATGGGAGAGGAG |
| GbRboh5/18-OE-R | GGGGACCACTTTGTACAAGAAAGCTGGGT GCCGTTAAAAATTTTCTTTGTGGAATTCG |
